# Supplementary material for: Peroxymonosulfate Activation by Fe@N Co-Doped Biochar for the Degradation of Sulfamethoxazole: The Key Role of Pyrrolic N
Source: Int J Mol Sci. 2024 Sep 30;25(19):10528. doi: 10.3390/ijms251910528 (PMC11477339; doi:10.3390/ijms251910528)
Supplement: Supplementary file 1 [file ijms-25-10528-s001.zip › ijms-3217751-supplementary.pdf]

Supplementary Material

Peroxymonosulfate activation by Fe@N co-doped BC for the degradation of sulfamethoxazole: The key role of pyrrolic N

Tong Liu[a,b], Chen-Xuan Li[a,b], Xing Chen[a,b], Yihan Chen[a,b], Kangping Cui[a,b,*], Dejin Wang[c], Qiang Wei[c,d,*]

[a] School of Resources and Environmental Engineering, Hefei University of Technology, Hefei 230009, P.R. China

[b] Key Laboratory of Nanominerals and Pollution Control of Higher Education Institutes, Hefei University of Technology, Hefei 230009, P.R. China

[c] School of Resources and Environment, Anqing Normal University, Anqing 246011, P.R. China

[d] CAS Key Laboratory of Urban Pollutant Conversion, Department of Environmental Science and Engineering, University of Science and Technology of China, Hefei 230026, P.R. China

[*]Corresponding Author.

E-mail address: cuikangping@hfut.edu.cn (K.P. Cui)

weiqiang07@ustc.edu.cn

Full postal address: School of Resources and Environmental Engineering, Hefei University of Technology, No. 193 Tunxi Road, Hefei 230009, P.R. China

Department of Environmental Science and Engineering, University of Science and Technology of China, No. 96 Jinzhai Road, Hefei 230026, P.R. China

Tel: +86 0551 62901541; +86 0551 63492298

Reagents and chemicals

The chemical reagents employed in this work include sulfamethoxazole (SMX, 98.0%, Rhawn), tetracycline hydrochloride (TC, 96.0%, Rhawn), 4-chloro-3-methyl phenol (CMP, 99.0%, Rhawn), norfloxacin (NOR, 98.0%, Rhawn), acetaminophen (ACT, 98.0%, Rhawn), sodium thiosulfate ($Na_2S_2O_3$, 99.0%, Rhawn), *L*-histidine (*L*-his, 98.0%, Rhawn), *tert*-Butanol (TBA, ≥99.5%, Rhawn), methanol (MeOH, ≥99.5%, Rhawn), and *p*-Benzoquinone (*p*-BQ, ≥99.0%, Rhawn), furfuryl alcohol (FFA, ≥98.0%, Aladdin), hydrochloric acid (HCl, 36.0−38.0%, Rhawn), sulfuric acid (H_2SO_4, 98.0%, Rhawn), sodium hydroxide (NaOH, 97.0%, Rhawn), sodium chloride (NaCl, 99.5%, Aladdin), monopotassium phosphate (KH_2PO_4, 99.0%, Aladdin), sodium bicarbonate ($NaHCO_3$, 99.8%, Aladdin), humic acid (HA, 98.0%, Aladdin), peroxymonosulfate ($KHSO_5 \cdot 0.5KHSO_4 \cdot 0.5K_2SO_4$, Oxone, Aladdin), 5,5-Dimethyl-1-pyrroline-N-oxide (DMPO, 97.0%, Rhawn), 2,2,6,6-tetramethyl-4-piperidinol (TEMP, ≥98.0%, Aladdin). All chemicals were employed without further purification and deionized water (18.2 MΩ/cm) was used throughout the experiments.

Characterization and analysis methods

Morphologies of Fe@N co-doped BC were observed by the scanning electron microscope (SEM, Hitachi SU8020) and high-resolution transmission electron microscope (HR-TEM, JEOL JEM-2100). The crystalline structure of Fe@N co-doped BC was detected by the X-ray diffractometer (PANalytical, Netherlands). ATR-FTIR spectra were obtained by a FT-IR spectrometer (Thermo, Nicolet 6700) in the 4,000−500 cm^{-1} range. The chemical states of Fe@N co-doped BC were analyzed by X-ray photoelectron spectroscopy (XPS, Thermo, ESCALAB250Xi). The SSAs and pore size distributions were analyzed with the Brunauer-Emmett-Teller (BET) method (Autosorb-IQ3, Quantachrome, USA). Raman spectrometer (HORIBA JOBIN YVON, LabRAM HR Evolution) was used to acquire Raman spectra.

SMX concentration was determined by high-performance liquid chromatography (HPLC, Shimadzu, LC-20AT). The mobile phase was SMX of acetonitrile/deionized water (0.1% formic acid) (70%/30%, v/v) at a flow-rate of 1.0 $mL \cdot min^{-1}$. The wavelength and column temperature were set to 255 nm and 30°C, respectively. The decomposition rate of PMS was determined by an ABTS colorimetric method (Wu et al., 2020). Total organic carbon (TOC) was measured using a Shimadzu TOC-vcph analyzer (Multi N/C 3000). The zeta potential of Fe@N co-doped BC was determined with a Zetasizer NanoBrook Omni (Brookhaven). Radical studies were performed with a JES-FA200 (JEOL) spectrometer. The intermediates were detected with ultra-performance liquid chromatography to quadrupole time-of-flight mass spectrometry (UPLC-TOF/MS, ACQUITY UPLC LCT Premier XE, USA).

Theoretical calculations

All DFT calculations are performed in Gaussian 16 software, using B3LYP/6-31 G (d, p) basis sets for optimization and transition state search for all configurations. All configuration optimization is carried out without symmetry restriction, and vibration analysis is performed after optimization to ensure no phantom frequency. Vibration analysis is performed on all transition state structures to ensure that there is only one virtual frequency, and that the virtual frequency corresponds to the direction of vibration to connect the reactants and products. The adsorption energy of AB structure is calculated based on the formula $E_b = E(AB) - E(A) - E(B)$, and the Gibbs free energy variation (ΔG) is also obtained under the method B3LYP/6-31G (d, p).

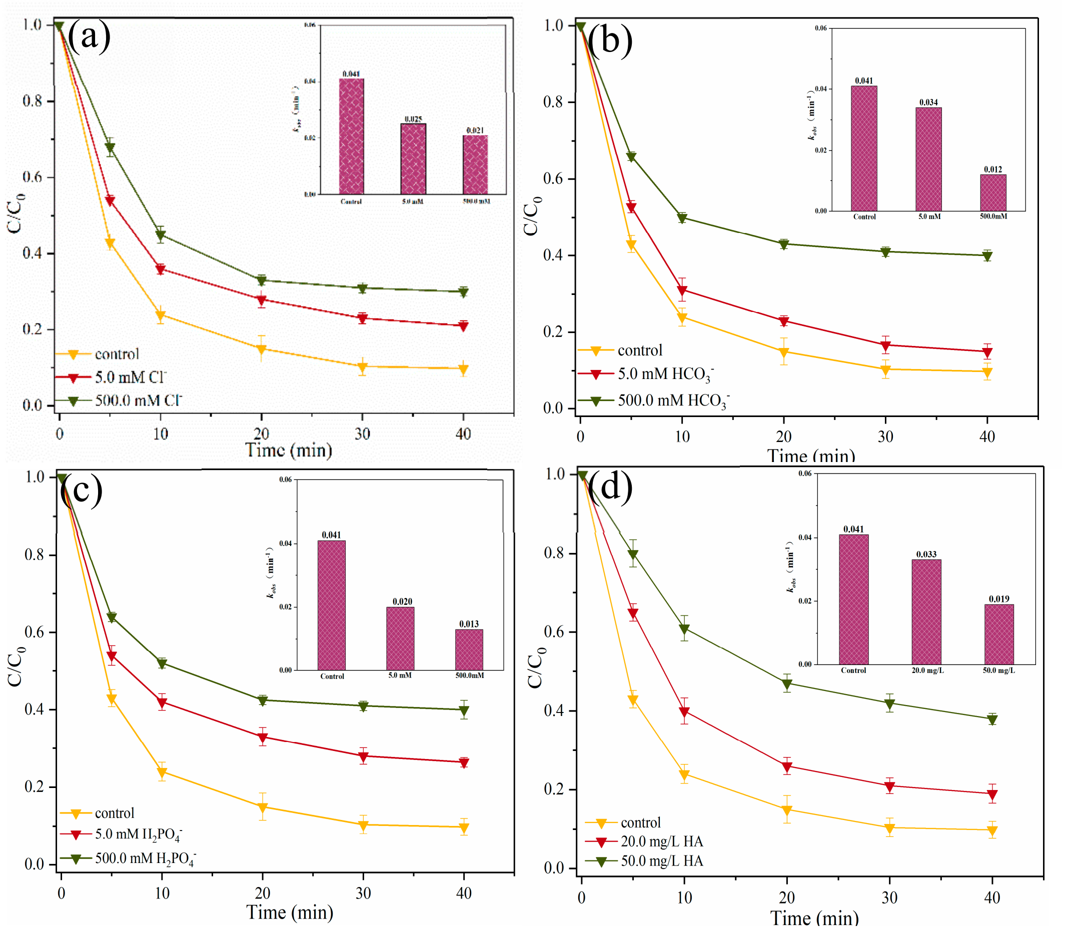

Fig. S1. Effects of Cl^- (a), HCO_3^- (b), $H_2PO_4^-$ (c) and HA (d) on SMX degradation. (Conditions: $[SMX]_0 = 10.0$ mg/L, [Fe@N co-doped BC] = 0.4 g/L, $[PMS]_0 = 0.6$ mM, $pH_0 = 7.0$, T = 25°C).

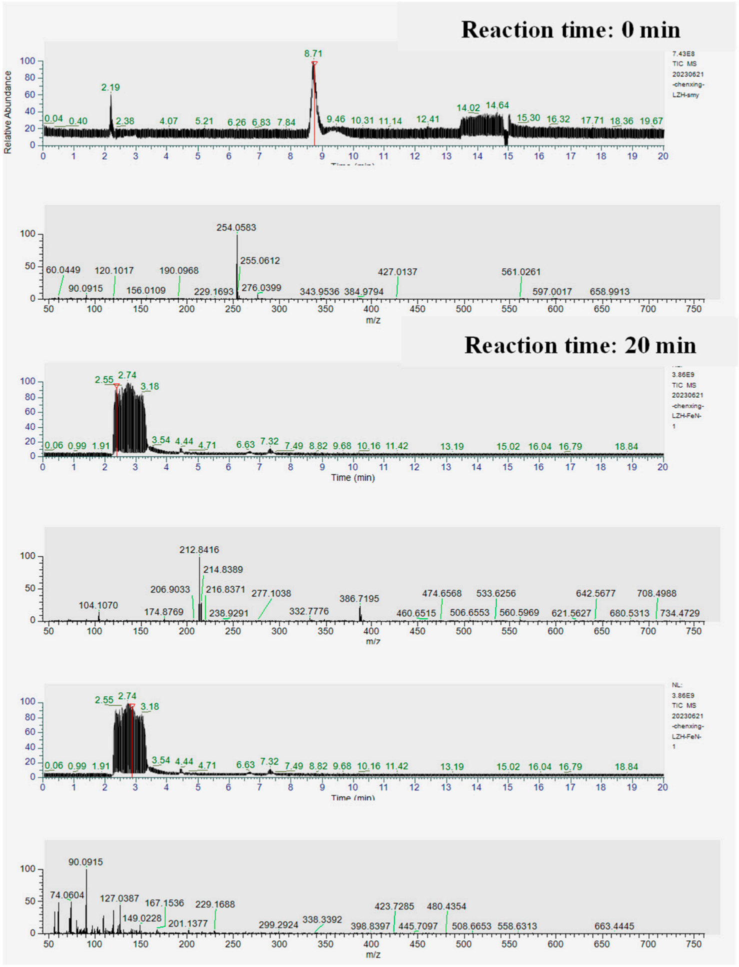

Fig. S2. Mass spectra of intermediate products.

Table S1. Comparison of the k_{obs} and R^2 of different systems.

Systems	k_{obs} (min^{-1})	R^2
BC+PMS	0.026	0.95
Fe-doped BC+PMS	0.028	0.95
N-doped BC+PMS	0.027	0.97
Fe@N co-doped BC+PMS	0.041	0.90

Table S2. Studies of the typical pollutants degradation with PS activated by biochar-based catalysts.

PPCPs	Catalyst	Oxidant source	Performance
20.0 mg/L	0.02 g/L CoS/BBC	0.3 g/L PMS	The degradation efficiency of SMX reached 99.12% within 10 min in CoS/BBC/PMS system (Huang et al., 2022).
10.0 mg/L	0.2 g/L Co@RBC_{800}	0.5 mM PMS	The optimal Co@RBC_{800} can degrade 100% of LVF in 10 min with a k of 0.331 min^{-1} (Liu et al., 2022).
5.0 mg/L	0.05 g/L EGB-900	4.0 mM PS	SMX can be completely removed within 90 min at EGB-900 concentration of 0.05 g/L and the k_{obs} value was 0.0655 min^{-1} (Qi et al., 2020).
20.0 mg/L	0.3 g/L γ-Fe_2O_3@BC	5.0 mM PS	A complete removal of BPA was obtained within 20 min with an observed rate constant (k_{obs}) of 0.1849 min^{-1}, which was almost two times as large as that (0.0956 min^{-1}) of pure biochar (Rong et al., 2019).
20.0 mg/L	0.2 g/L Fe-N-BC	1.0 mM PS	In the Fe-N-BC/PS system, almost all acid orange (AO7) was removed within 90 min with an apparent rate constant (k_{obs}) of 0.114 min^{-1} (Li et al., 2020).

Table S3. The contribution of each reactive species.

Systems	•OH	$SO_4^{\bullet-}$	$O_2^{\bullet-}$	$^1O_2$	Degradation rates/%
Fe@N co-doped BC/PMS	√	√	√	√	90.2
Fe@N co-doped BC/PMS+MeOH	-	-	√	√	33.4
Fe@N co-doped BC/PMS+TBA	-	√	√	√	73.5
Fe@N co-doped BC/PMS+*p*-BQ	√	√	-	√	84.7
Fe@N co-doped BC/PMS+*L*-his	√	√	√	-	73.2
Computation	16.7%	40.1%	5.5%	17.0%	-
Relative contribution	21.1%	50.2%	7.2%	21.5%	100.0%

Table S4. Main elements of the obtained catalysts.

Samples	C (at. %)	N (at. %)	O (at. %)	Fe (at. %)
BC	65.3	0.2	24.0	5.0
Fresh Fe@N co-doped BC	47.6	6.1	31.0	15.3
Used Fe@N co-doped BC	39.6	5.3	35.0	20.1

Table S5. Intermediates of SMX degradation detected by UPLC-TOF/MS.

Structural formula	Molecular formula	m/z
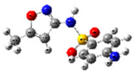	$C_{10}H_{11}N_3O_3S$	284
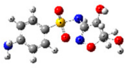	$C_{10}H_{12}N_3O_5S$	288
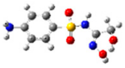	$C_8H_{11}N_3O_4S$	246
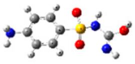	$C_7H_9N_3O_3S$	216
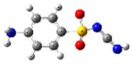	$C_7H_7N_3O_2S$	198
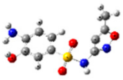	$C_{10}H_{10}N_3O_4S$	270
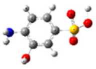	$C_6H_7NO_4S$	190
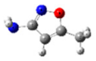	$C_4H_6N_2O$	99
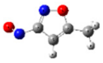	$C_4H_4N_2O_2$	111
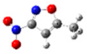	$C_4H_4N_2O_3$	127

References

Huang, X., Yu, Z., Shi, Y., Liu, Q., Fang, S., 2022. Highly efficient activation of peroxymonosulfate by Co, S co-doped bamboo biochar for sulfamethoxazole degradation: Insights into the role of S. Journal of Environmental Chemical Engineering 10(5).

Li, X., Jia, Y., Zhou, M., Su, X., Sun, J., 2020. High-efficiency degradation of organic pollutants with Fe, N co-doped biochar catalysts via persulfate activation. J Hazard Mater 397, 122764.

Liu, J., Jiang, J., Wang, M., Kang, J., Zhang, J., Liu, S., Tang, Y., Li, S., 2022. Peroxymonosulfate activation by cobalt particles embedded into biochar for levofloxacin degradation: Efficiency, stability, and mechanism. Separation and Purification Technology 294.

Qi, Y., Ge, B., Zhang, Y., Jiang, B., Wang, C., Akram, M., Xu, X., 2020. Three-dimensional porous graphene-like biochar derived from Enteromorpha as a persulfate activator for sulfamethoxazole degradation: Role of graphitic N and radicals transformation. J Hazard Mater 399, 123039.

Rong, X., Xie, M., Kong, L., Natarajan, V., Ma, L., Zhan, J., 2019. The magnetic biochar derived from banana peels as a persulfate activator for organic contaminants degradation. Chemical Engineering Journal 372, 294-303.

Wu, Z., Wang, Y., Xiong, Z., Ao, Z., Pu, S., Yao, G., Lai, B., 2020. Core-shell magnetic Fe3O4@Zn/Co-ZIFs to activate peroxymonosulfate for highly efficient degradation of carbamazepine. Applied Catalysis B: Environmental 277.
